# High-Dose Benzodiazepine Dependence: A Qualitative Study of Patients’ Perceptions on Initiation, Reasons for Use, and Obtainment

**DOI:** 10.1371/journal.pone.0142057

**Published:** 2015-11-10

**Authors:** Michael Liebrenz, Marcel Schneider, Anna Buadze, Marie-Therese Gehring, Anish Dube, Carlo Caflisch

**Affiliations:** 1 Department of Psychiatry, Psychotherapy and Psychosomatics, Psychiatric Hospital, University of Zurich, Zurich, Switzerland; 2 Department of Forensic Psychiatry, Institute of Legal Medicine, University of Bern, Bern, Switzerland; 3 Department of Surgery, Division of Visceral and Transplantation Surgery, University Hospital, Zurich, Switzerland; 4 Ulmenhof, Sozialtherapie, Ottenbach, Switzerland; 5 University of Pennsylvania Health System, Philadelphia, Pennsylvania, United States of America; University of Manitoba, CANADA

## Abstract

**Background:**

High-dose benzodiazepine (BZD) dependence is associated with a wide variety of negative health consequences. Affected individuals are reported to suffer from severe mental disorders and are often unable to achieve long-term abstinence via recommended discontinuation strategies. Although it is increasingly understood that treatment interventions should take subjective experiences and beliefs into account, the perceptions of this group of individuals remain under-investigated.

**Methods:**

We conducted an exploratory qualitative study with 41 adult subjects meeting criteria for (high-dose) BZD-dependence, as defined by ICD-10. One-on-one in-depth interviews allowed for an exploration of this group’s views on the reasons behind their initial and then continued use of BZDs, as well as their procurement strategies. Mayring’s qualitative content analysis was used to evaluate our data.

**Results:**

In this sample, all participants had developed explanatory models for why they began using BZDs. We identified a multitude of reasons that we grouped into four broad categories, as explaining continued BZD use: (1) to cope with symptoms of psychological distress or mental disorder other than substance use, (2) to manage symptoms of physical or psychological discomfort associated with somatic disorder, (3) to alleviate symptoms of substance-related disorders, and (4) for recreational purposes, that is, sensation-seeking and other social reasons. Subjects often considered BZDs less dangerous than other substances and associated their use more often with harm reduction than as recreational. Specific obtainment strategies varied widely: the majority of participants oscillated between legal and illegal methods, often relying on the black market when faced with treatment termination.

**Conclusions:**

Irrespective of comorbidity, participants expressed a clear preference for medically related explanatory models for their BZD use. We therefore suggest that clinicians consider patients’ motives for long-term, high-dose BZD use when formulating treatment plans for this patient group, especially since it is known that individuals are more compliant with approaches they perceive to be manageable, tolerable, and effective.

## Introduction

To varying degrees, benzodiazepines (BZDs) exhibit amnestic, sedative, anticonvulsant, and muscle-relaxant effects. These effects are believed to result from BZDs’ properties as modulators of GABA_A_ receptors, binding to the chloride-channel molecular complex and thereby enhancing the effects of GABA by increasing chloride (Cl−) flux and frequency of channel opening that ends in hyperpolarization and neuronal inhibition [1–3]. The specific properties of BZDs have led to their wide use in general clinical practice, as well as in psychiatry for the treatment of anxiety and mood disorders, insomnia, states of withdrawal, delirium, management of acute agitation or aggression, and catatonic syndrome [4–6]. Furthermore, BZDs are used in anesthesiology for anesthesia induction and maintenance, prior to diagnostic or therapeutic interventions; and in neurology for the treatment of acute prolonged epileptic seizures (duration ≥ 2min) [7–10]. BZDs have well-documented effectiveness over the last 50 years [11] and remain one of the most commonly prescribed medication classes by practitioners for various indications [12–14], despite their side effects [15, 16]. The development of dependence is characteristic of BZDs and typically follow long-term high, normal, or even low-dose use [17]. The definitions for “long term use” have varied widely over the years, ranging from 6 weeks [18] to greater than one year [19, 20]. Prevalence of BZD use in the general population is estimated to be between 2.0–7.4% with data suggesting that 14.1–76% of this BZD using population are long term (more than 4 to 6 months) users [21–24]. Although data on “high-dose” users is not readily available, a study from Switzerland estimated that 1.6% of the Swiss adult population uses doses, exceeding at least twice the maximum recommended daily dose [12]. According to some authors, however, current diagnostic manuals have not reliably defined BZD dependence [25], and they argue for two [17] or three subgroups, respectively [26]. The first group consists of patients who become dependent as a result of regular, repeated prescriptions of BZDs for an underlying disorder such as for sleep or anxiety, but continue to use within the recommended doses. A second group is comprised of individuals who begin with the use of physician-prescribed BZDs and subsequently progress to inappropriate use and increase their dosage excessively. The third group consists of individuals who were never prescribed BZDs and use them for recreational purposes (e.g., enhancing or alleviating the effects of other psychotropic substances, reducing withdrawal symptoms, or seeking a euphoric effect by using high doses). [26].

Although these three groups may overlap, little quantitative or qualitative evidence exists to support this categorization. Some studies have investigated the reasons for continued use of BZDs among patients with repeated prescriptions while in treatment with a general practitioner [27], but qualitative exploration of these clinical issues in individuals with high-dose, long-term and/or otherwise inappropriate use of BZDs (e.g. mixing, repeated dose escalation, euphoric effect enhancement) and illicit use (e.g. illegal acquisition, criminal activities) remains scarce. This is problematic because not only is this latter group reported to suffer more severe mental disorders [28] and is less likely to achieve long-term abstinence by recommended discontinuation strategies [29, 30], but also because it is increasingly understood that treatment interventions that do not take subjective experiences and beliefs into account are unlikely to enhance social or medical help-seeking behavior and may therefore have a negative impact on overall treatment outcome [31–33]. For these reasons, the present study aims to develop the literature on self-perceived reasons for initiating and continuing BZD use, as well as obtainment strategies among high-dose BZD-dependent patients.

## Materials and Methods

### Design of study and participants

We conducted an exploratory qualitative study among adult patients meeting criteria for (high-dose) BZD-dependence according to the 10th revision of the International Classification of Diseases (ICD-10)[34]. To the authors’ knowledge there is no universally accepted definition of high-dose benzodiazepine dependence. In previous studies on detoxification for high dose benzodiazepine dependence, patients with a dose of 40 to 500 mg diazepam equivalents (Median 150mg) were included [35]. Quaglio et al included in their study on “High dose benzodiazepine dependence: Description of 29 patients treated with flumazenil infusion and stabilized with clonazepam” patients that received a daily dose (converted to diazepam equivalents) that ranged from 38 to 1800 mg per day (median 333 mg/day)[36]. In a previous publication [30] we were reluctant to describe ‘high-dose’ users as a well-defined group (e.g. by a certain amount of diazepam equivalents) as, in our opinion, this does not reflect clinical reality and may provoke unnecessary dose–range discussions. We chose to use the following definition for that study, which also formed the basis for the present manuscript:

Patients who typically have a high-dose, long-term and/or otherwise problematic use of benzodiazepines, such as mixing benzodiazepines (e.g. midazolam, flunitrazepam, lorazepam, oxazepam), escalating their dosage repeatedly, using benzodiazepines to enhance the effects of other substances, obtaining their BZDs by illegal means and those who experience negative social consequences. These included high-dose users as defined by the use of 40 mg diazepam equivalents per day over an extended period of time and/or otherwise problematic use of benzodiazepines, such as mixing, repeated dose escalation, euphoric effect enhancement, or illegal acquisition strategies.

The participants in this study were recruited from patients who presented to the Psychiatric University Hospital, Zurich, between 03/2011 and 11/2012, using a combination method of purposeful- and saturation sampling principles. To achieve greater variation of themes and motives, we recruited subjects from general treatment settings as well as from specialized units for the treatment of substance-use disorders. Patients that were recruited from the outpatient units of the Psychiatric University Hospital were seen on an “as needed” basis by their physicians. Treatment duration (weeks to years) and form of intervention varied amongst participants, ranging from an abstinence oriented benzodiazepine discontinuation approach to the more permanent prescription of slow-onset, long-acting BZDs. Furthermore, the sample was chosen to incorporate diversity with regards to: (a) past clinical experience and comorbidity, (b) duration of high-dose benzodiazepine use, (c) gender, (d) age and (e) occupational status. Exclusion criteria were insufficient language skills and acute intoxication.

The research team contacted sixty potential participants in person, previously identified by treating physicians as those patients who had a problematic (high-dose) use of benzodiazepines, but not for those fulfilling only ICD-10 criteria for BZD dependence. Potential participants were verbally informed of the reasons for the present research and received an opt-in letter (384 words). Nineteen declined to participate. Barriers to participation were directly addressed in a few instances. Two potential participants declined inclusion in this study, because they felt the amount of honorarium (a 5 Swiss Franc gift card for inpatients and a 5 Swiss Franc cash payment for outpatients) was insufficient. More commonly, potential participants left the impression of being too ashamed to discuss the subject (14). In three cases, potential participants initially agreed to be interviewed, but then withdrew their consent for participation during the interview–citing a lack of interest in the research topic. In total, 41 subjects provided their written, informed consent and completed the interview. The full chart of each patient, including their complete biographical and psychiatric history and their diagnosis according to ICD-10, was provided by the clinic.

### Data collection and interview

To ascertain participants’ self-perceptions and experiences in relation to: (1) the reasons for their initial and continued use of BZDs, and (2) the the circumstances surrounding their BZD obtainment, we conducted single, unstructured, in-depth interviews lasting between 60–90 minutes. One female and one male researcher conducted the interviews, who were at the time of the study employed (MG) by, or associated with, the Psychiatric University Hospital (MS). Data was collected outside the regular treatment setting (e.g. hospital-ward), in an atmosphere that allowed participants to feel free to fully express their own views and perceptions, but within the in- or outpatient clinics (Lenggstrasse 32 and Selnaustrasse 9, Zurich, Switzerland). Participants were assured that no personally identifiable information from the interviews would be made available to treating psychiatrists, psychologists and/or third parties (such as referring physicians or law enforcement personal). During the interviews, only the participant and interviewer were present in the room. Participants were provided with mineral water during the interview and could take breaks as needed.

MG had a degree in psychology (Master of Science UZH/MSc UZH) while MS was a medical student in his sixth and final year. All interviewers were trained by ML and CC to conduct interviews. The research team had gathered previous experience employing qualitative research methodology in patients with mental disorders and co-occurring substance use disorders. Results have been reported elsewhere [37–39].

All interviews began with narrative opening questions, but a self-developed topic guide provided a flexible framework to explore beliefs that were not spontaneously covered in participants’ initial comments. The topic guide is presented in Table 1. Furthermore, probes were used to explore the reasons for BZD use if these did not arise spontaneously in patients’ initial narratives. Throughout the period of the interviews, the research team met regularly to discuss the data, as well as the focus of subsequent interviews.

**Table 1:** Topic guide.

**Main questions**
« Can you tell me about the first time you used a benzodiazepine? »
« Why do you think people use benzodiazepines? »
« How do you feel about using benzodiazepines ?»
« What do you experience when you use a benzodiazepine? »
« How did you get benzodiazepines (back then and now) ? »
« Have you ever thought about stopping benzodiazepines ? »
« Were there times when you stopped using benzodiazepines ? »
« Have you ever thought about how benzodiazines influence your body ? »
« (…) »
**Additional questions**
« Did you notice any changes (in your symptoms) when you were using benzodiazepines ? »
« How do you feel about your physicians benzodiazepine prescribing practices ? »
« Were you able to follow his/her dosage recommendations? »
« (…) »
**Clarifying questions**
« Can you expand a little on this »
« Can you tell me anything else »
« Can you give me some examples »

In addition, we allowed themes and motives identified in earlier interviews to be explored in those that followed, combining the principles of maximum variation and complexity reduction to simultaneously widen the scope of results and examine previous assumptions.

There were no repeat interviews. Transcripts were not returned to participants; however, interviewers paraphrased and summarized the main points with participants during the interviews. Prior to publication of the manuscript, participants did not provide feedback on the overall themes and findings.

### Data analysis

All interviews were digitally recorded using dictamus for iOS, and then transcribed verbatim from Swiss German (an Alemanic dialect spoken in the “German-Speaking” parts of Switzerland) into Standard German, since Swiss German is not a written language. Analysis of data and coding was done manually using a word processor (Microsoft Word for Mac 2011).

Any personally identifying information was removed from the transcript and transcripts were assigned a code number. Mayring’s qualitative content analysis approach was used to evaluate findings [40]. This meant allowing the data to "speak for itself,” rather than approaching the data from within existing assumptions. According to Mayring “the main idea of the procedure of analysis is thereby, to preserve the advantages of quantitative content analysis as developed within communication science and to transfer and further develop them to qualitative-interpretative steps of analysis” [41, 42]. Materials were coded using an inductive qualitative procedure. Categories obtained were discussed among the research team (ML, AB, MG, CC) to validate ratings and achieve consensus. ML applied the final code, with confirmation of consistency through the blind dual coding of transcripts with MG and CC. Recruitment of participants continued until we had reached saturation of the data, operationally defined as when no new themes emerge and we had tested all the categories for disconfirming case variations. Prior to submission of this manuscript categories, codes and representative quotations were translated into English by ML and proofread by a native English speaker (Corinna Fales of New York). English transcripts were then made available to the research team to control for accuracy. During the review process of the original manuscript, concerns about the syntax of individual quotations were raised. Where possible quotes were grammatically corrected to improve readability.

Authorization by the local ethics committee “Cantonal Ethikkommission Zürich” (www.kek.zh.ch) was obtained before the study was conducted (E-23_2009). All participants were assured complete confidentiality, and their written informed consent to the study, and specifically to the digital recordings of the interviews were obtained. The ethics committee approved this consent procedure.

We reported this study using COREQ guidelines [43].

## Results

41 participants completed the one-on-one in depth interviews. The participants were predominantly male (75.6%). The mean age of the participants was 39.5 years ± SD 9.2 (median 39.0 years). At the time of the interview participants were using benzodiazepines for a self-reported mean time of 8.2 years ± SD 6.82 (median 5.0 years). The mean diazepam equivalent dosage (maximum, self-reported) was 83 mg ± SD 69 (median 70 mg, range 10 mg– 300 mg). Three participants were included despite using diazepam equivalents of only 10mg/d because they fulfilled other inclusion criteria: obtaining BZDs by illegal means and experiencing negative social consequences.

The vast majority of participants (75.6%) suffered from a variety of psychiatric comorbidities (chart-verified). To compensate for an initial overrepresentation of individuals who used multiple substances (past or current use of one (19.5%) or more (70.7%) psychotropic substances), we looked for potential interviewees without (9.8%) and repeatedly requested attending psychiatrists to pass on opt-in letters to their patients so that we could obtain a maximum variation sample and make our findings more generalizable. Socioeconomic status varied: 12 (29.3%) participants were employed at the time of interview (unskilled, semiskilled and skilled), while 11 (26.8%) were unemployed or reliant on social welfare and 17 (41.4%) received some form of disability pension or were retired. Therefore, this sample represents–as intended following our sampling approach–a heterogeneous group of individuals.

Participants’ demographic characteristics, benzodiazepine consumption patterns and psychiatric comorbidities are described in Table 2.

**Table 2:** Participants’ demographic characteristics, benzodiazepine consumption patterns and psychiatric comorbidities.

Characteristics	Number (%) of participants (Total N = 41)
**Gender**	
Male	31 (75.6)
**Age group (year)**	
< 26	3 (7.3)
26–35	11 (26.8)
36–45	16 (39)
46–55	10 (24.4)
> 55	1 (2.4)
**Current employment status** ^**a**^	
Working	12 (29.3)
Unemployed (or Social Welfare)	11 (26.8)
Disability pension (or Retired)	17 (41.4)
Missing data	1 (2.4)
**Age at intial benzodiazpine use** ^**a**^	
< 19	10 (24.4)
19–25	6 (14.6)
26–35	13 (31.7)
36–45	9 (22.0)
45 >	2 (4.9)
Missing data	1 (2.4)
**Diazepam equivalent dosage** ^**b**^	
< 40 mg	9 (22.0)
40–79 mg	17 (41.4)
80–120 mg	6 (14.6)
>120 mg	9 (22.0)
**Number of benzodiazepines used** ^**c**^	
1	3 (7.3)
2–3	22 (53.6)
>3	16 (39.0)
**Duration of regular benzodiazepine use (year)** ^**d**^	
< 1	4 (9.7)
1–3	9 (22.0)
4–5	9 (22.0)
6–9	4 (9.7)
>9	14 (34.1)
Missing data	1 (2.4)
**Inpatient discontinuation attempts** ^**d**^	
< 1	1 (2.4)
1–3	24 (58.5)
4–9	9 (22.0)
>9	7 (17.0)
**Intital benzodiazepine obtainment** ^**a**^	
General practioner	10 (24.4)
Psychiatrist	7 (17.0)
Other medical speciality	3 (7.3)
Friends, colleagues and/or black market	16 (39.0)
Other	3 (7.3)
Missing data	1 (2.4)
**Past and current strategies for benzodiazepine obtainment** ^**a**^	
Legal and illegal strategies	27 (65.8)
Only legal	13 (31.7)
Missing data	1 (2.4)
**Lifetime substance use other than benzodiazepines** ^**e**^	
Alcohol	26 (63.4)
Cannabis	15 (36.6)
Heroin	28 (68.3)
Stimulants	26 (63.4)
LSD	3 (7.3)
None	4 (9.8)
**Lifetime psychiatric comorbidities except for substance use disorders** ^**f**^	
Schizophrenia, schizoptypal and delusional disorders	1 (2.4)
Mood [affective] disorders	21 (51.2)
Neurotic, stress-related and somatoform disorders	12 (29.3)
Disorders of personality and behaviour in adult persons	14 (34.1)
Behavioural and emotional disorders with onset usually occurring in childhood and adolescence	1 (2.4)
None	10 (24.4)

^a^ as of the time of interview, self reported, where possible chart verified.

^b^ maximum dosage of a benzodiazepine that were ever used (expressed in diazepam equivalents), self reported, where possible chart verified.

^c^number of benzodiazepines used during lifetime self reported, where possible chart verified.

^d^ as of the time of interview, chart verified.

^e^ multiple answers possible, self reported, where possible chart verified.

^f^ according to ICD-10, as of the time of interview, multiple answers possible, chart verified.

### Participants’ view of circumstances surrounding their initial and continued use of BZDs

All participants developed explanatory models for their initial use of BZDs. In most cases, this use was regarded by participants as problematic at the time of the interview. We identified four main categories and listed them in order of importance based on the number of respondents. Although some overlap occurred, no categories applied to all participants.

#### Use of BZDs to cope with symptoms of psychological distress or mental disorder other than substance use

When participants were asked about the initial circumstances under which they began using BZDs, they often described stressful life events that had resulted in a deterioration of their emotional or psychological wellness, or directly reported symptoms of their mental disorder (frequently naming a diagnosis) that had led them to the use of these substances.

Participants commonly linked their initial use of BZDs with symptoms of *sleep disorder*, such as difficulty falling asleep, staying asleep, and/or waking abruptly during the night with feelings of terror:

“*I have not been taking tablets all my life*. *Once I could not fall asleep and then my ex-boyfriend gave me a Dormicum® (midazolam) and told me*: *“Take this if you want to be able to sleep*!*”… So I started with Dormicum®*…*“*

*VP 21*, *female*, *age 47*

The feelings of *anxiety*, like fear, worry, uneasiness, or *compulsiveness* (as obsessive, distressing, intrusive thoughts and behaviors) were also described.

*“Certainly the strong obsessive behavior*, *which I have noticed in early childhood and the anxiety which I felt…and my first GP he recognized this and I was very happy and relieved and he was also very generous to dispense a bottle of 100 tablets of Lexotanil® 5 mg (bromazepam)…”*

*VP 11*, *male*, *age 49*

Social anxiety was commonly mentioned in this context, and participants often said that BZDs relieved their symptoms promptly and reliably, as compared to other treatment options:

*“Yes*, *initially because of my sociophobia and erythrophobia*, *other prescription drugs were tried*, *but they did not ‘hit it off’…and then we just stayed with Temesta® (lorazepam)…and then I went a lot with my nurse into the city*, *going shopping a little*, *just among the people*, *exposure therapy…but the effort it takes to go through a day when suffering from sociophobia without prescription drugs is just extremely taxing*. *And then I just got tired of it*, *to put so much effort into everyday things like going to COOP (grocery store) and this is when I noticed that it would not work without drugs*.*”*

*VP 06*, *male*, *age 30*

Frequently, subjects described a combination of all of the above symptoms that ultimately led to their initial use of BZDs. This perception is captured by the statement of participant VP 04, a 35-year-old woman who had been a professional painter but was receiving a disability pension at the time of the interview:

*“I have known benzodiazepines for around seven years*. *I got them in the beginning of a depression*, *because of strong anxiety*, *sleeplessness*, *and restlessness*, *which left me unable to concentrate…and it helped pretty good*. *I did not get very tired and I was not lying around in bed all day…I took it to achieve stability*, *to dampen anxiety and to improve sleep…”*

*VP 04*, *female*, *age 35*

Participants who experienced symptoms in the wake of adverse life events, and linked the onset of their symptoms and their initiation of BZD use to these circumstances, further illustrate this explanatory approach. This was showcased in the interviews with participant VP 28, a 39-year-old woman working as a welder.

*“…then I got laid off*. *It had all just gotten too much for me*, *and then I found a new position that I did not like*, *actually where I did not like anything*, *and then I could not sleep anymore and I started to feel anxious and panicky when I stepped on the train (to take me to work)…and then I always took one in the morning and at half-past seven*, *another one…”*

*VP 28*, *female*, *age 39*

While participants perceived BZDs having *antidepressant properties*, and framed this as an explanation for starting BZDs, it was difficult to distinguish from euphoria-seeking as a rationale for use, and probably represents a combination of both.

*“…I had a phase in which I was exhausted and beaten*. *She then diagnosed a ‘latent depression’ and prescribed BZDs to me…and I have to say it really helped me in the beginning…effects were good*. *That is the devilish thing*. *This stuff stimulates*, *and you forget your problems*, *you are a little bit in a different world…”*

*VP 25*, *male*, *age 32*

Some participants drew comparisons between BZDs and other forms of pharmaceutical interventions for their underlying disorder, pointing out that they were started on BZDs because other (non-benzodiazepine) drug treatments (e.g. antipsychotics) had proved insufficient:

*“In 2005*, *so some six years ago*, *I got in touch with benzodiazepines for the first time*. *I was treated on an inpatient ward because of a schizoaffective disorder*. *Leponex® (clozapine) was deemed insufficient by the treating physician*. *That is why I was prescribed 1 mg Temesta® (lorazepam) four times a day*. *So*, *one in the morning*, *one in the afternoon*, *one in the evening*, *and one at night*. *That worked very well*. *That is the curse and mercy of these damn benzos*: *that they work so well*.*”*

*VP 29*, *male*, *age 50*

Less frequently were participants reporting that they initiated BZDs for their amnestic properties:

*“…this is where I was followed by a stalker*. *That was a really troublesome guy*. *He followed me for several weeks and this and that*, *and he could be really nice once*, *but then he started to threaten me—that I must marry him*, *and then the sentence that so many guys say*: *“You love me*, *you just do not know it yet*.*” It all started to wind up*, *and that just killed my psychologically*, *and then I started to buy benzos on the black market*, *in order to forget this thing*. *Well*, *you can never really forget*, *only bear it*.*”*

*VP 13*, *female*, *age 42*

#### Use of BZDs to cope with symptoms of physical or psychological discomfort associated with a somatic disorder

Multiple participants associated their initial use of BZDs with experiencing symptoms of somatic disorders. The predominant reason cited by participants was pain-related symptoms leading to sleeplessness:

*“So my problems started with pain*. *I could not sleep*, *developed a sleeping disorder*, *because I woke up as a result of pain*. *I then went to a physician*, *who started me on Stillnox® (zolpidem)*. *After some time*, *around three months it did not work and we switched to Seresta® (oxazepam)…and when that did not work we went to Dormicum® (midazolam)”*

*VP 35*, *male*, *age 47*

*“The first time I got benzos and used them was when I was 18 years old*. *My ex-boyfriend was dealing them*, *not just benzos*, *but drugs in general*. *And the first time I had a really strong toothache and I could not fall asleep*, *he said*: *“Come on*, *inject one Dormicum® (midazolam)*, *then you will be able to sleep*. *This is how it started…”*

*VP 24*, *female*, *age 22*

Other reasons included neurological symptoms, such as tremor or anxiety, and depressive symptoms linked to an invasive surgical intervention:

*“It started some six years ago*. *I had an essential tremor*, *that is why I received a beta blocker*, *but in a very low dose*. *My doctor told me that it should soon work without it*. *But soon I got a terrible headache and blood-red*, *inflamed eyes*. *And I was trembling like crazy*. *I increased the beta blocker*, *but that did not help*. *I increased even further without any improvement*. *Then I went to a neurologist who prescribed Xanax® (alprazolam) to me*. *That was fantastic*, *in the beginning…”*

*VP 15*, *male*, *age 50*

*“I started with the antidepressant (participant regarded BZDs as antidepressants) one month prior to my tumor surgery*. *It was the first time I received an antidepressant*. *I had a tumor in my cerebellum*, *the diencephalon and the ear…I was scared that after the surgery I would be paralyzed and my children would have another image of their mother*. *I told my doctor that I would rather die than to change that way*. *I then started with antidepressant drugs*, *initially with Temesta® (lorazepam)…”*

*VP 33*, *female*, *age 51*

#### Use of BZDs to cope with symptoms of substance-related disorders

Numerous participants took a different approach when asked about their view of the reasons for their initial BZD use. They attributed their use to the management of undesirable effects of other licit or illicit psychotropic substances. These participants associated their use of BZDs less for recreational purposes, and more to alleviate euphoric and agitated states, and to attenuate alcohol and opioid withdrawal symptoms. These subjects frequently linked their first use of BZDs with a desire to change their consumption patterns of other substances, believing BZDs were less harmful than other substances, such as alcohol or cocaine. Furthermore, they viewed BZDs as a controlled, pure substance, without the risks of poisoning or contamination inherent in “street drugs”.

Most often, participants in this category started using BZDs in the context of alcohol withdrawal. Interestingly, long-term BZD use was not just perceived as a consequence of a failed alcohol withdrawal approach:

*“Actually*, *it really started with an alcohol withdrawal*. *This is how I began using them*, *since benzos were used for withdrawal*. *And after that I just got stuck to that dose*, *never really completing to taper them out…”*

*VP 02*, *male*, *age 38*

But was viewed also as a less hazardous and self-initiated form of self-medicating. The statement of Mr. VP 20, a 54-year-old former teacher and travel guide, best exemplifies this explanatory model:

*“Back then*, *especially alcohol was a problem*. *I drank responsibly on many days*, *too much on others*. *By trying to get off alcohol*, *I suggested to my GP that I could try in the future to reduce my feelings of inner tension with benzodiazepines*, *without having to reach for alcohol*. *This is when he began to prescribe benzos to me*. *I used them for a long time just complementary or sporadically*, *because I am often very nervous or have sweaty palms…”*

*VP 20*, *male*, *age 54*

Subjects also frequently reported that they had started using BZDs to “calm down” from the stimulating effects of cocaine:

*“I used cocaine*. *And to calm down*, *I always found them very beneficial*. *And additionally I noticed that you could completely unwire*. *And it was good for sleeping*. *I could sleep very well*.*”*

*VP 30*, *male*, *age 37*

They perceived BZDs to be less dangerous and some viewed it as clean and cheap, and providing the same euphoric effects as street drugs:

*“…Because I thought and …ehm…I just did not know it…I viewed it as something harmless*, *compared to sugar (heroin) and cocaine*. *And also when you were on a lot of cocaine*, *it calmed you down*…*”*

*VP 03*, *male*, *age 48*

*“…Benzos were never a problem back then*, *not like heroin*. *Everything shifted more into a direction*, *that what I really wanted was cocaine*, *cocaine*, *and more cocaine*. *And to calm down*: *benzos*. *Eventually I noticed that I prefer just to take benzos*. *They are clean*, *are less costly*, *and have for me the same effect as cocaine…and this is how I started with Dormicum® (midazolam)*.*”*

*VP 31*, *male*, *age 43*

Participants with a history of opioid dependence often reported that they began using BZDs to attenuate symptoms of heroin withdrawal and that BZDs functioned as substitutes until they could obtain opioids again. Furthermore, these individuals linked their first experiences with BZDs to the open drug scene in Zurich, and thus to a time in which Opioid Maintenance Treatment was not as readily available as it is today:

*“…So initially I took heroin… when the effects wore off*, *there was no way I could sleep*. *So instead of taking heroin*, *I rather take medicine*. *That did not just save money*, *but it was also better to fall sleep…and so almost because of the financial considerations*, *I switched to benzodiazepines*.*”*

*VP 22*, *male*, *age 46*

*“…The reason why I personally became a Dormicum® (midazolam) addict is that the material on the street is really bad at the moment*. *And as a heroin addict…over the holidays*, *Christmas*, *and New Year*, *for example*, *it was impossible to find anything*. *I then tried Dormicum® (midazolam) and discovered that the flash of Dormicum® and alcohol is almost identical to that of heroin*.*”*

*VP 23*, *male*, *age 44*

*“…Because in principle heroin was the problem*. *Benzodiazepines were fill-in if there was no heroin at hand…and because I noticed that it alleviates withdrawal*. *And because I got the most impossible ideas how I could obtain heroin again (during withdrawal)…”*

*VP 31*, *male*, *age 43*

#### Use of BZDs for recreational purposes and social reasons

Some participants repeatedly commented on the recreational effects of BZDs. Participants who had also used other psychotropic substances and had discovered the paradoxical effects of BZDs, either accidentally or from friends and colleagues, predominantly adopted this explanatory model for initial BZD use.

On the one hand, this motive included the enhancement of positive feelings and the search for stimulating, euphoric and amnestic effects:

*“It is around 10*, *12*, *or 13 years ago*, *I took half a tablet*, *since it was a gray day and I was bored*. *It gave me euphoria*. *I already knew that it was possible to develop an addiction*, *but I thought it would never happen to me and I could control it…”*

*VP 12*, *female*, *age 66*

*“It was at the time of the Letten (the second open drug scene in Zurich)…This is back at the time when there were still Rohypnol® 2 mg (flunitrazepam)*, *the white ones*, *available*. *It is almost 20 years ago*. *Initially I just took half of the 2 mg tablet*, *so 1 mg*. *And I sat down and I thought*, *‘Nothing happens*, *no flash*.*’ When I got back up*, *I had a hard time doing so and also with my balance*. *That's when I thought*: *“Cool*, *this does flash*,*” and I found that pretty funny…”*

*VP 40*, *male*, *age 40*

*“The first time I got in touch with benzos*, *I was around 18 years old*. *I had a girlfriend back then*. *I did not know that she was doing drugs*, *hard drugs*. *That is when I took Rohypnol® (flunitrazepam) the first time*. *A friend of mine gave us the tip to try half a pill*. *We did not notice anything…so we thought*: *‘Let us take the other half*, *too*.*’ We were just lying around like dead flies…”*

*VP 39*, *male*, *age 40*

And on the other hand, it included the reasons related to sensation-seeking and fitting in the social setting.

*“Back then I was living in a staff dormitory of Roche Pharmaceuticals and during the week I was living in Basel and we were smoking cannabis here and there*. *Professionally*, *we were all very interested in psychoactive substances; 80% of the class were interested and tried things*. *I noticed then that Rohypnol® (flunitrazepam) did good things for me*, *that I was not anxious*, *that I could better concentrate*. *I found myself being easy (to get along with) and relaxed…”*

*VP 17*, *male*, *age 45*

*“Uii*, *I am really not supposed to say that*. *Ah well…That was back…I was around 12 or 13 years old*. *And my aunt*, *she had them prescribed…and my cousin had such a strange friend and since we did not have anything to smoke*, *to blaze*, *he then suggested to take the Rohypnol® (flunitrazepam) ah Valium® (diazepam) from my aunt…and I knew from my mother*, *that when she is not doing well*, *so that she could sleep…*

*VP 05*, *male*, *age 32*

### Patient’s past experiences, activities and strategies for obtaining BZDs

With few exceptions, participants spoke very openly about their past experiences obtaining BZDs. We identified three major legal, illegal, and mixed strategies for acquiring them:

#### Participants’ past experiences and views on legal or official strategies for obtainment

Interviewees most commonly received BZDs legally, through dispensing physicians’, by prescriptions filled at drug stores, at inpatient and outpatient treatment centers, or through the jail and prison system.

*“Legal*, *and this for the 22 years I am in Switzerland*. *I don't feel well*, *I go to a doctor*, *he prescribes drugs*, *then I go with the prescription to the drug store…”*

*VP 18*, *male*, *age 45*

*“The first time I got it prescribed was in jail… because I was a little stupid*, *I told them I was taking different stuff and blew it completely out of proportion*.*”*

*VP 05*, *male*, *age 32*

“Doctor shopping”, that is to switch from a physician with a restrictive approach toward BZDs to a physician with more lenient views, was a common strategy for obtainment as was visiting several doctors simultaneously:

*“Yes*, *and these benzos are so addictive*. *When one doctor tells you*: *‘This is it*, *no you had enough*, *no*, *you have to discontinue them*,*’ then you start doing funny things like just looking for another doctor*. *And then you begin even making downright nonsense*, *like going to one doctor who prescribes you something and then additionally to another who dispenses them to you*.*”*

*VP 04*, *female*, *age 35*

Within this context participants’ frequently described a tendency to exaggerate current BZD usage and to give false or misleading information on the number of physicians from whom they were receiving these substances:

*“If I want a higher dosage*, *I can play a little with the doctor*. *That means I usually take 30 mg Valium® (diazepam) but then I go to the doctor and tell him*: *“I take 80 mg per day”*. *And then he will start me on 80 mg per day*. *Then I can start with that dose*. *I do play with the doctors to get a higher dose (…)*. *And the doctor will think that I take that amount and fill out a prescription*.*”*

*VP 01*, *male*, *age 38*

#### Participants’ experiences with illicit and mixed strategies for acquiring BZDs

When obtaining BZDs illegally, they were usually obtained (1) in the black market by people dealing them, (2) directly from drug stores without a prescription, by forging prescriptions or claiming the identity of physicians, (3) through friends and colleagues who had received BZDs legally or were themselves in a position to obtain them, or—(4) in one case—directly from the manufacturer. Participants repeatedly described more difficulty obtaining fast-onset, short-acting BZDs by legal means and attributed their higher street value to more “drug-like” effects. This became especially apparent for flunitrazepam (Rohypnol*®*) which is a controlled-substance according to Swiss legislation.

*“I bought it on the street*. *In the beginning 1*, *2 a day*, *later 8 then 15…I partially dealt with it*, *selling it on*.*”*

*VP 32*, *male*, *age 36*

*“…had a connection*. *It does not really matter what kind of connection*. *And there I could get it at invoice 54*.*70 Swiss Francs for the box of 100 and this is how I made business*.*”*

*VP 30*, *male*, *age 37*

*“I just went to the drug stores and asked for it*. *They gave it to me without a prescription and I just went from one drug store to another*. *That was my entire day*.*”*

*VP 12*, *female*, *age 66*

*“My physician never prescribed Dormicum® (midazolam) to me*. *Just sometimes he made out a prescription afterwards*, *so that I did not have to pay for it when I had gotten it from a drug store*. *Sometimes I called in his name in the drug store and told him they should give Mr*. *X (the patient) a box of Dormicum® (midazolam); he would fax the prescription*. *And then I left my phone number and when they did call back I answered in his name and told them they could go ahead and give Mr*. *X Dormicum (midazolam)®*.*”*

*VP 31*, *male*, *age 43*

*“I called my brother*. *He is an anesthesiologist and has access to benzos*. *I begged him*: *“Please*, *please reduce my despair*, *it is driving me up the wall*.*”…He then brought me two pills*, *Temesta® (lorazepam) and two pills Xanax® (alprazolam)…and in the middle of benzodiazepine withdrawal I went to my mother*. *I knew that she had benzos too…*

*VP 20*, *male*, *age 54*

*(How did you get these drugs*? *Could you just enter the warehouse of Roche and take it*?*) “Yes*, *we had this stuff by the kilo*.*”*

*VP 17*, *female*, *age 45*

While it was possible to differentiate between these approaches, it became evident that the vast majority of our study subjects had been going back and forth between legal and illegal ways of obtainment, regardless of their initial reasons for using BZDs, whether due to mental or physical disorder or for recreational purposes.

*“In the canton Graubuenden*, *I once had a doctor where I could go once a week and he would dispense it to me*. *However*, *I always had to pay for it*. *Later*, *I went to the canton Zurich*, *where I just got it on the street…And then partially with a doctor but more so on the street*, *because the amount the doctor was giving to me did not suffice my needs*.*”*

*VP 31*, *male*, *age 43*

Interestingly, however, perceptions of the role of prescribing physicians depended on the method of initial BZD use. Those who initially received BZDs from their physicians for psychological or physical distress tended to view those doctors negatively:

*(indignantly) “No*, *(I never obtained them on the street)*, *but I still claim that the neurologist I had back then in the University hospital was a dealer*. *This is how he tied patients to himself*. *He gave me so much*, *in the beginning a prescription for 200 Xanax® (alprazolam)*, *that when I went to the drug store they did not want to fill it…I think it is his scam…with benzos he ties his patients…”*

*VP 11*, *male*, *age 49*

*“…and then later I noticed that he is not a real doctor*. *He is too busy and does not want to help me to stop or to taper out benzodiazepines*. *And later I thought*, *he just wants my money and he does not care about my health and my problems…and*, *sadly*, *though all the doctors and psychiatrists in Switzerland hand out these drugs too easily…and all that is business*.*”*

*VP 01*, *male*, *age 38*

In contrast, individuals who began self-medicating with BZDs to alleviate the side effects of substance-related disorders, or for recreational purposes, more often identified doctors as individuals who tried to support them:

*“It was always difficult to find someone who would prescribe Dormicum® (midazolam)*. *I then had my GP*. *With him I got along really well*. *He knows me since early on*. *He then supported me and told me he would rather give it to me before I would do shit again*.*”*

*VP 34*, *male*, *age 31*

*“…I get along really well with him (my physician)*. *And he told me*: *‘Better like this*, *than that you get criminal ideas*.*’ (On the street) it costs 40–50 Swiss Francs for 10 pills*. *And with my doctor everything went through health insurance…”*

*VP 07*, *male*, *age 41*

*“I simply told him (the physician) I need Dormicum® (midazolam)*, *I am addicted and that if he would not prescribe it to me I would have to go buy it illegally*, *on the black market*.*”*

*(And then he was prescribing it to you*?*)*

*“Yes*, *quite simply*, *I was just honest with him*.*”*

*VP 26*, *male*, *age 35*

While participants clearly preferred to have physicians prescribe BZDs to avoid engaging in criminal activity, reduce their risk of consuming contaminated pills, and lower their costs, many of them had experienced treatment termination over suspected abuse of this substance.

*“No*, *I did do that sometimes (buy them on the black market) but that is not exactly ideal*, *because you never know what you get*. *They tell you it is this*, *but in fact it is that*. *Then you buy even more*. *At the end you are left with no money and do not have anything*. *To receive it from a doctor is undoubtedly the best thing*.

*VP 35*, *male*, *age 47*

*“My doctor does not prescribe me benzodiazepines anymore*. *She told me that I would not get any benzos from her (…)*. *The only option left is back on the street…That*, *I did not want*.*”*

*VP 25*, *male*, *age 32*

*“Close to the Stadelhofen train station*, *I found a doctor*. *I told him that I wanted to withdraw from heroin and I would need benzos for that*. *So I received two packages from him*, *every two weeks or so… After a while he said that the withdrawal should be finished*. *And he just simply stopped it*. *Then you suddenly have nothing…so I went back onto the street…”*

*VP 40*, *male*, *age 40*

In this context some participants felt that treatment termination not only forced them into the black-market for acquisition but was associated with a loss of control and further increase in usage. The statement of VP 13, a former commercial clerk in a bank, typifies this perception:

“But with this benzos (prescribed from GP) I could handle it, I was sticking to his regime. How he prescribed it–I took it. After that (termination) I went back to the black-market and there I started with other benzos and that is when it slipped from my hands.”

*VP 13*, *female*, *age 42*

## Discussion

This exploratory study highlights the beliefs and perceptions of high-dose, long-term BZD- dependent patients in regard to their initial drug use and the reasons for their continued use, as well as their obtainment strategies and related experiences.

Four themes were identified as factors contributing to participants’ initial use of BZDs. The first theme, *BZD use to cope with symptoms of psychological distress or mental disorder other than substance use*, underscores the finding that the majority of participants perceived symptoms of sleep disorder, restlessness, nervousness, anxiety, depression, or another mental disorder as a major distress requiring additional help. Frequently, participants reported that their GP or psychiatrist then initiated pharmaceutical treatment with BZDs. Other subjects received BZDs the first time from friends or colleagues because they complained about discomfort—and only later appealed to their physicians, who in some cases began prescribing the medication. Management of perceived sleep disturbances was the most frequently named reason for initial BZD use, whether or not subjects suffered from a co-morbid substance-use disorder.

These findings are consistent with an earlier study of British long-term BZD users (with a median consumption duration of five years), which reported that 61% of subjects claimed that they initially received BZDs from their general practitioner for insomnia, followed by anxiety, depression and muscle tension [44]. Our findings are also similar to a more recent study that additionally reported that certain medical conditions, alcohol and other substance withdrawals, stress, and coping with domestic violence were reasons given by interviewees for their initial use of BZDs [27]. However, our findings are in contrast to a survey of BZD users in a Methadone Maintenance Treatment Program, that found curiosity (46%) to be the main reason for BZD use, followed by relieving tension (41%) and feeling good (37%) [45].

The second theme, *benzodiazepine use to cope with symptoms of physical or psychological discomfort associated with somatic disorder*, was most often associated with sleep deprivation because of pain and with neurological symptoms such as tremors. The negative perceptions of some of our subjects further illustrate the need for providing comprehensive patient information on the potential benefits and risks of habit-forming substances. This is particularly true when considering treatment for somatic disorders [46–48]. Even though current practice parameters by the American Academy of Neurology have a Level B recommendation for alprazolam for the treatment of essential tremor, it is also important to consider the elevated risk for abuse and dependence [49, 50] [51].

In our sample, motives from the third theme, *benzodiazepine use to cope with symptoms of substance related disorders*, were more frequently cited than were motives from the fourth theme, *benzodiazepine use out of sensation seeking and for recreational purposes*. This finding suggests that participants with a substance-use disorder primarily use BZDs not for recreational effects, but to alleviate agitated states and attenuate alcohol and opioid withdrawal symptoms. Interestingly, subjects perceived BZDs as less dangerous, and cleaner, and uncontaminated, as compared to other legal and illegal substances: in their view, BZDs protected against street-drug-related hazards and therefore constituted a harm-minimization strategy.

While previous studies have reported deliberate recreational and thrill-seeking use [17, 45, 52], the use of BZDs to reduce harm is currently under-investigated. We further find it noteworthy that participants with an opioid dependence often reported having begun BZDs use to attenuate symptoms of heroin withdrawal, and linked it to a time when opioid maintenance treatment (OMT) was not readily available. This finding suggests that BZD dependence in this group could have partly been avoided by offering low-threshold access to treatment [53, 54]. Although it has been suggested that this reason for initiating BZD use has decreased in importance because of the increased availability of OMT in Switzerland over the last decades [55, 56], it might still be a significant factor for patients elsewhere who have less access to maintenance therapy [57].

In this sample of high-dose-dependent participants, the theme of BZD use as a stand-alone drug to induce euphoric effects, enhance positive feelings and create stimulation was not as common as expected [26, 45]. We suspect that the high number of interviewees with co-occurring mental disorders and correspondently severe symptoms, contributed to the preference for medically related explanatory models.

Unsurprisingly, participants had developed multiple strategies for obtaining BZDs, and these included legal as well as illegal means. In this sample, BZD acquisition through more sophisticated channels such as the Internet was not a factor [58]. However, it became very clear that the majority of participants had gone back and forth between legal and illegal means of obtainment, irrespective of their reasons for use, and in most cases for several years. It was also apparent that participants viewed prescription practices as varying greatly between physicians, which is consistent with previous reports from the United Kingdom [27].

Our study suggests that patients have a clear preference for staying in treatment with a physician, both for financial and for health-related reasons, and that they often experience immediate treatment termination when their BZD dependence is discovered. On the other hand, some participants reported that their physicians initiated BZD treatment or continued prescribing BZDs to avoid further harm even when they were aware of an abuse or dependence problem. Essentially, they chose not to follow current recommendations for a complete BZD withdrawal in dependent patients [59], and instead adopted a maintenance approach [30].

Although the street value of BZDs does not always make rational sense [17] and is influenced by availability as well as reputation [60], we found that fast-onset, short-acting substances such as Dormicum® (midazolam) and Rohypnol® (flunitrazepam)—the latter falling under controlled-substance legislation in Switzerland—were considered more desirable by recreational users and were perceived to have a higher resale value.

In this sample of 41 (high-dose) benzodiazepine dependent patients we did not find qualitative evidence for a categorization into the three subgroups of benzodiazepine dependence according to initiation and progression of use, as Ashton et al suggested [26]. We found significant similarity, among those who started using them with or without a prescription, casting doubt on whether (treatment) interventions based on such categorization would indeed be clinically helpful. Our findings instead indicate that when treating this patient group, it might be necessary to intensify communication between different physicians involved in the management of the same patients, as well as with dispensing drug stores. This is not with the aim of ending treatment, but to detect patterns of high dose use earlier and to modify the treatment approach as indicated. For example, in primary care specialties, involving the pharmacist more actively resulted in an improvement of functioning and quality of life in the management of hypertension, diabetes and asthma [61–63]. Elsewhere we proposed a provisional stepped care approach for high-dose benzodiazepine dependent patients who wish to discontinue benzodiazepines and find themselves unable to so [30]. The narratives of this patient group against the background of duration of use and number of unsuccessful detoxification attempts, underlines the need for such an undertaking [16, 64].

Despite its strengths, our study has limitations to consider. Most participants in this study were in treatment for their BZD-dependence at the time of the interview, a large proportion had been receiving BZD’s from physicians and were participating in a study conducted by physicians and psychologists. These contextual factors increase the likelihood that medical explanations for behavior were elicited from participants. Furthermore data on benzodiazepine consumption patterns are self-reported, relying on an active reconstruction process called recall, and may have resulted in a recall bias. Although the interviews were conducted outside the treatment setting and subjects were informed that no information except suicidal ideation would be made available to the treating physicians, some participants may have offered responses they believed were being sought by the interviewer. However, considering the fact that participants readily described the methods of their BZDs acquisition, we have reason to believe that the majority truthfully reported their own perceptions. The study is also subject to selection bias, where it is likely that some individuals chose not to participate due to shame or other discomfort about their BZD use [65]. Although we aimed for balance in gender, in this study there was an under-representation of females (10 female vs 31 male), who may have differing perceptions on initiation, reasons for use, and obtainment of benzodiazepines. Moreover, this study focused on high-dose BZD users only, so it is unclear to what extent the present findings can be generalized for patients with other patterns of use.

## Conclusions

We addressed two areas of research that focused on patients with high-dose BZD dependence and elicited their perceptions of: (1) the reasons they initiated and continued their use of BZDs, and (2) how they obtained the drugs. We identified a multitude of reasons that perpetuated BZD use, including psychological distress and symptoms of mental disorder (e.g., substance use and/or somatic illness; recreational and social reasons). Irrespective of comorbidity, participants had a clear preference for medically related explanatory models. Subjects with a previous history of use and/or abuse of multiple substances often viewed BZDs as less dangerous and associated their use more frequently with harm-reduction measures than with recreational purposes or social reasons.

Although specific obtainment approaches varied widely, most participants oscillated between legal and illegal strategies, often relying on the black market when faced with treatment termination.

We suggest that clinicians consider our findings while planning treatment for this heavily burdened patient group, since it is known that individuals are more compliant with treatment approaches they perceive as manageable, tolerable, and effective [66].
